# Feasibility of Utilizing Wastewaters for Large-Scale Microalgal Cultivation and Biofuel Productions Using Hydrothermal Liquefaction Technique: A Comprehensive Review

**DOI:** 10.3389/fbioe.2021.651138

**Published:** 2021-11-19

**Authors:** Sourav Kumar Bagchi, Reeza Patnaik, Ramasare Prasad

**Affiliations:** ^1^ Department of Bioscience and Bioengineering, Indian Institute of Technology Roorkee, Roorkee, India; ^2^ DBT-IOC Centre for Advanced Bioenergy Research, Research and Development Centre, Indian Oil Corporation Limited (IOCL), Faridabad, India

**Keywords:** bio-crude, biofuel, hydrothermal liquefaction, microalgae, raceway ponds, wastewaters

## Abstract

The two major bottlenecks faced during microalgal biofuel production are, (a) higher medium cost for algal cultivation, and (b) cost-intensive and time consuming oil extraction techniques. In an effort to address these issues in the large scale set-ups, this comprehensive review article has been systematically designed and drafted to critically analyze the recent scientific reports that demonstrate the feasibility of microalgae cultivation using wastewaters in outdoor raceway ponds in the first part of the manuscript. The second part describes the possibility of bio-crude oil production directly from wet algal biomass, bypassing the energy intensive and time consuming processes like dewatering, drying and solvents utilization for biodiesel production. It is already known that microalgal drying can alone account for ∼30% of the total production costs of algal biomass to biodiesel. Therefore, this article focuses on bio-crude oil production using the hydrothermal liquefaction (HTL) process that converts the wet microalgal biomass directly to bio-crude in a rapid time period. The main product of the process, i.e., bio-crude oil comprises of C16-C20 hydrocarbons with a reported yield of 50–65 (wt%). Besides elucidating the unique advantages of the HTL technique for the large scale biomass processing, this review article also highlights the major challenges of HTL process such as update, and purification of HTL derived bio-crude oil with special emphasis on deoxygenation, and denitrogenation problems. This state of art review article is a pragmatic analysis of several published reports related to algal crude-oil production using HTL technique and a guide towards a new approach through collaboration of industrial wastewater bioremediation with rapid one-step bio-crude oil production from chlorophycean microalgae.

## Introduction

Renewable energy is typically defined as the energy derived from natural resources and is naturally replenished continuously. Renewable energy resources include solar, wind, rain, tidal waves, geothermal heat, and bioenergy. Renewable energy resources are found over a wide range of geographical regions throughout the world as compared to the fossil fuel resources that are available only in very few countries. A report of Renewable Energy Policy Network for the 21st century (REN Report, 2014; REN Report, 2015), the global investments in renewable technology sectors have been calculated to be US$214 billion. The countries like United States, China, Norway, and Denmark have invested a lot in solar, hydro, wind, and biofuels production sectors (REN Report, 2014; Mathiesen et al., 2015). It is therefore essential to discuss the importance of bioenergy, a kind of renewable energy, derived from various biological sources to generate heat or to produce the liquid transportation fuels often coined as biofuels. As per an analysis of the scenario in the recent years, biofuels are among the most widely used renewable energies that provide approximately 9–10% of the global primary energy supply (IEA Report, 2013). NS news agency (NS Energy Report, 2019) has reported that 2,616 thousand bbl day^−1^ of biofuels were produced in the world during 2019. The United States followed by Brazil have conquered the biofuels market with a collective share of approximately 87% of the world’s production. The United States is the primary biofuel producer in the world with a total production of 1,190 thousand bbl day^−1^. The country has harnessed ∼46% of the world’s biofuel production in the year of 2018. It is also to be mentioned that United States is the world’s top biodiesel producer with a total share of 19%, equivalent to 136.2 thousand bbl day^−1^ in 2018 (https://www.nsenergybusiness.com). These countries and few more, have diverted their attention towards biofuel production from green chlorophycean “microalgae” (Galadimaa and Muraza, 2018). The use of microalgae for biofuel production has numerous advantages over the other biodiesel production sources, and hence, these microalgae are often coined as the “Green gold” for biofuel production (Bagchi et al., 2018).

The major advantages of using microalgae for biofuel production are enumerated below:

Biofuel derived from the grains and oil seeds have a large land and carbon imprint, instigating the food vs. fuel argument (Mandal and Mallick, 2009). Microalgal biomass with a much faster growth rate has a usual capability to bio-mitigate CO_2_ while trapping sunlight with an efficiency of 10–50 times higher than the common terrestrial plants (Li et al., 2008), Microalgae are capable of producing higher amounts of oil (58,700–136,900 L ha^−1^ year^−1^) per unit area of land as compared to other oil-producing crops (Chisti, 2007). Comprehending such high oil yielding potentials of microalgae with effective CO_2_ bio-fixation up to 12–15% in air mixture, the locally isolated green microalga *Scenedesmus obliquus* was shown to be a potent alternative as a renewable source for biomass production with a maximum biomass yield of 7.01 g L^−1^ under mixotrophic cultivations in photobioreactors under controlled culture environment (Bagchi and Mallick, 2016). As microalgae are aquatic, the microalgae can be cultivated in freshwater, sea-water, brackish-water, or even various wastewaters (de Godos et al., 2009). Microalgae are used for the algal biorefinery studies by sequentially extracting many important compounds (Patnaik and Mallick, 2015; Patnaik et al., 2019). After the extraction of bio-oil from microalgae, the algal biochar can be used as an enhancer of soil fertility along with preventing soil degradation through efficient carbon sequesteration in the soil.

However, despite these advantages use of microalgae for biofuel production is yet to be cost-competitive with fossil-based fuel due to the upstream and downstream challenges such as high cost of nutrients, energy-intensive harvesting, drying, lipid extraction, and transesterification techniques. One of the methods to counter these problems is large scale cultivation for enhanced biomass production (Patnaik and Mallick, 2015; Severo et al., 2019). The large-scale mass cultivations of microalgae in raceway ponds are well established by several researchers. The large-scale practice in raceways had started in the early years of 1950s by cultivating the green microalga *Chlorella* (Brennan and Owende, 2010) and was in full operational phase in the late 1960 using the “high rate algal ponds (HRAP)” with Oswald’s large-raceway-pond designs (Oswald and Golueke, 1960). Microalgae mass cultivation in raceway ponds has now been considered as the most promising means for large scale biomass production in terms of less capital investment and low running cost compared to the engineered photobioreactor (PBR) systems. Basically, photobioreactors are useful to maintain the monoalgal culture but the overall operational cost is extremely high as compared to the open large-scale raceway ponds (Bagchi and Mallick, 2016; Bagchi et al., 2019). One of our recent studies has already shown that an annual biomass productivity upto a high value of 13.12 tons ha^−1^ year^−1^ can be achieved if cultivated for ten cultivation cycles per annum. The study was conducted in four numbers of 40,000 L capacity raceway ponds by Bagchi et al. (2019). In another study by us at the same geographical location, it was observed that *Scenedesmus accuminatus* organism produced a biofuel yield of 2.14 tons ha^−1^ year^−1^ in open raceway pond batch cultivation (Koley et al., 2019). However, contrary to this Zhang et al. (2018) reported a comparatively lower biofuel yield of 0.79 tons ha^−1^ year^−1^ algal biofilm raceway ponds.

Nowadays, waste disposal is a worldwide problem. In the current scenario, waste discharges from various industries and city sewages are the primary sources of water pollution. Conventional wastewater treatment systems do not seem to be the definitive solution to pollution and eutrophication problems. Secondary sewage treatment plants are specifically designed to control the number of organic compounds in wastewaters. However, pollutants, mainly nitrogen, phosphorus, sulfur are only slightly affected by this type of treatment. Wastewater treatment by microalgae using the wastewater or waste disposal as the growth medium for large-scale algal cultivations is possibly the best way to solve these tailbacks effectively (Mallick et al., 2016).

Another serious challenge for the upscaling of biodiesel production is the exploitation of various low-energy intensive harvesting and drying techniques and the development of cost-effective lipid extraction methods. Removal of water from the wet algal biomass is necessary for prolonged storage of the feedstock and carry out further downstream processes like lipid extraction followed by biodiesel production. Generally, wet microalgae contain ∼90% moisture. The drying technologies are typically utilizing high extensive heat energy, which puts a significant obstacle to the microalgal biodiesel market assessment on a profitable basis (Lardon et al., 2009; Patil et al., 2011; Bagchi et al., 2015). It is also a well-known fact that the key constraint for downstream process of microalgal biodiesel production is the enormous expenditures associated with the extraction of lipids followed by the transesterification process. There are a lot of researches being carried out for developing the lipid extraction processes from microalgae. The Folch method (Folch et al., 1957) and the Bligh and Dyer (Bligh and Dyer, 1959) technique are the most acclaimed and commonly practiced total lipid extraction protocols for microalgal biodiesel production also adopted for large-scale extraction processes (Kumar et al., 2015). These techniques are performed by using a considerable volume of solvents as chloroform: methanol: 2:1. The modified method of the above for the extraction of all lipids classes was suggested by Matyash et al. (2008) in which Methyl-tert-butyl ether (MTBE) was utilized as a solvent. The method proved to be successful in the extraction of almost all lipids classes to portray entirely accurate lipidomic profiles.

From the above discussions, it is now well understood that despite numerous independent research works on microalgal cultivation, wastewater remediation and HTL, a workable strategy combining all the three factors for reducing the economic gap between fossil-based and biomass-based fuels is not available to the extent of our knowledge. Therefore, we in this review report intend to categorically discuss the important points reported in various research works related to algal cultivation using wastewaters and propose a strategy for “waste to wealth” generation combining microalgal growth and lipid accumulation with wastewater bioremediation followed by HTL technique for deriving bio-crude oil directly from wet algal biomass, thereby recommending a synergistic approach for sustainable biofuel production.

## Wastewater Utilization for Microalgal Cultivation

### Laboratory Based Studies

It is prominent that microalgae can bioremediate wastewater by the removals of ammonium, nitrate, nitrite, and phosphate from a variety of wastewater sources (Mallick, 2002). Various Researchers reported that the microalgae could grow in different kinds of wastewaters, and the wastewater resources are proved to be the best potential source of cost-effective biofuel production (Woertz et al., 2009). However, the wastewater utilization to enhance algal growth, thereby low-cost lipid production, and the exploration of microalgae’s pollutant removal efficiency is still a minimal approach in terms of outdoor large-scale algal culture exploitations.

The chlorophycean microalga *Scenedesmus obliquus* has shown an elevated biomass and lipid yield by utilizing the mixture of poultry litter and municipal secondary settling tank discharges in the amount of 15 g L^−1^ (Mandal and Mallick, 2011). The swine manure wastewater was successfully utilized for the cultivation of 97 microalgae obtained from algae-bank and 50 other algal strains isolated from the local waterbodies in Minnesota, United States of America. The maximum biomass yield was achieved up to 2.03 g L^−1^ for the locally isolated microalgal strain UMN 271 (Zhou et al., 2012). One report observed that the mixed microalgal consortium was cultivated in two phases comprising initial growth phase (biomass enhancement; 8 days) under mixotrophic mode using domestic sewage wastewater followed by temperature stressed starvation phase. The biomass yield was recorded high enough in this production process (Venkata Subhash et al., 2014). Another report also demonstrated that the microalga *Chlorococcum* sp. was grown in sea-water based saline medium supplemented with waste glycerol available from the biodiesel industries with a maximum biomass yield was 0.85 g L^−1^ (Beevi and Sukumaran, 2015) (Table 1). In continuation, the biomass yield were found significantly higher as 6.0 g L^−1^, for the mixed algal consortium cultivated with the dairy manure as a rich nutrient source (Table 1) (Chowdhury and Freire, 2015). The green microalga *Chlorella vulgaris* was grown under ammonia-rich wastewater (Markou, 2015). The utilization of wastewaters was also quite useful for algae cultivation as per the research work carried out in our lab. The microalga *Chlamydomonas debaryana* IITRIND3 was successfully cultivated in different wastewaters from domestic, sewage, paper mills, and dairy wastewaters, respectively. The maximum biomass yield was depicted as 3.66 g L^−1^ in dairy wastewater whereas 3.56 g L^−1^ in domestic wastewater, respectively (Arora et al., 2016). Biomass yield found in this process by utilizing the wastewaters was quite productive, and the yield values are significantly higher than many other reports published till date. In another study done in this laboratory, the crude glycerol (CG) was used as low cost by-product obtained from the biodiesel production process for the cultivation of the microalga Chlorella minutissima (MCC27), with the maximum biomass yield 3.13 g L^−1^, respectively (Table 1) (Katiyar et al., 2018). It was also demonstrated that the microalga *Chlorella* sp. was successfully cultivated in the aerated seafood processing wastewater for higher biomass accumulation, lipid production as well as the major nutrients’ removal from the wastewater. The study also has shown that the total nitrogen (TN) and total phosphorous (TN) contents in the wastewater were constantly decreased during the end of the cultivation period of the microalgae. The total nitrogen concentration was reduced to a deficient level of 4.11 mg L^−1^, which was only 3.4% of the initial concentration. Further calculations have also indicated that ∼93 and ∼50% of the eliminated nitrogen and phosphorous were assimilated by the alga during the end of the course of the investigation, showing that the tiny organisms “microalgae” are effectually the potential sources to utilize for removing the nitrogen and phosphorous from the wastewater bodies (Gao et al., 2018) (Table 1). Elystia et al. (2020) has reported that the green microalga *Chlorella pyrenoidosa* was successfully cultivated using palm oil mill effluent (POME) wastewater as a growth medium and the maximum specific growth rate was 0.306 days^−1^ with the highest number of cells was 3.530 × 10^7^ cells ml^−1^. However, in this experiment, the researchers have not specified the exact biomass yield or biomass productivity. The fresh water chlorophycean microalga *Scenedesmus pecsensis* was proved to be a potential agent of wastewater bioremediation by 68.2% phosphate and 49.3% nitrogen removal. The alga was cultivated using rice mill effluent wastewater as a low-cost medium. The biomass yield was also found to be quite higher as 5.3 g L^−1^ (Keerthana et al., 2020).

**TABLE 1:** Tabulations of various reports on elevated biomass yield by using cost-effective cultivations as the utilization of waste disposal and wastewaters.

Name of the microalga	Operational description	Maximum biomass yield (g L^−1^)	References
*Scenedesmus obliquus*	Poultry litter + municipal secondary settling tank wastewater discharges	2.0	Mandal and Mallick (2011)
Locally isolated microalga	Digested swine manure wastewater	2.03	Zhou et al. (2012)
Mixed microalgae culture	Mixotrophic mode using sewage wastewater followed by temperature stressed starvation phase	2.49	Venkata Subhash et al. (2014)
*Chlorococcum* sp. RAP13	Sea water-based medium, supplemented with biodiesel industry waste glycerol	0.85	Beevi and Sukumaran (2015)
Mixture of algae	Dairy manure as a nutrient source	6.0	Chowdhury and Freire (2015)
*C. vulgaris*	Ammonia-rich wastewater by using poultry litter	1.5	Markou (2015)
*Chlamydomonas debaryana* IITRIND3	Algal cultivation in different wastewaters as domestic, sewage, paper mills, and dairy wastewaters	3.66	Arora et al. (2016)
*Chlorella minutissima*	Crude glycerol (CG) used as low cost by-product	3.13	Katiyar et al. (2018)
*Chlorella* sp.	Seafood processing wastewater	1.55	Gao et al. (2018)
*Scenedesmus pecsensis*	Rice mill effluent wastewater	5.29	Keerthana et al. (2020)

### Utilization of Wastewaters for Large-Scale Microalgal Cultivation Systems

#### Basic Concept of Raceway Ponds

Scientists have urged on the essentiality of large-scale microalgae cultivation for commercial level biofuel production (Moazami et al., 2011; Moazami et al., 2012). There are two main types for large-scale microalgae cultivation, closed systems (photobioreactors) and open ones. A possible low-cost culture system strategy for bio-oil production on a commercial scale is the use of raceways ponds or circular tanks (Moheimani and Borowitzka, 2007; Ashokkumar and Rengasamy, 2012). Compared to the photobioreactors, raceway ponds are generally preferable for large-scale algal biomass production due to the significantly less capital investment and lower maintenance cost, utilization of wasteland or barren lands, and easy operation techniques (Chisti, 2008). It is also an essential factor that the microalgal cultivation inside the raceway ponds requires the optimum stirring for continuous or semi-continuous mixing to recirculate the microalgal culture (Chisti, 2016; Koley et al., 2019). However, there are many limitations of raceway pond culture of microalgae. Open raceways are more prone to contamination by other organisms such as bacteria, fungi, other microalgal starins, diatoms. Achieving elevated productivity and maintenance of mono-algal strain are the real shortcomings of cultivation in open raceway ponds (Bagchi et al., 2018). Therefore, it is very necessary to grow the desired algal starin in the raceways covered with polyhose made with thick transparant polythenes. This is also highly essential to protect the algae from the schorching sunrays particularly in the tropical regions where the temperature are above 45°C during the summer season. Several other essential parameters like pH, DO, light intensity, temperature, aerator speedflow must be monitor time to time for the optimum productivity in large-scale raceway ponds (Bagchi et al., 2019; Koley et al., 2019).

#### Summarization of Study Reports of Microalgal Cultivation With Wastewaters

The major disadvantages of using the conventional facultative algal ponds are the maintenance of monocultures, the requirement of various chemical flocculation techniques that are generally very costly processes for microalgal harvesting, and may not deliver regular and effective nutrients’ removal (Abdel-Raouf et al., 2012). In contrary to this, the use of shallow, paddle-wheel driven and high rated algal ponds can generate much more higher amount of algal biomass up to 30–40 tons ha^−1^ year^−1^ with a provision to explore the bioflocculation or self-flocculation techniques that may afford the cost-effective microalgal harvesting (Slade and Bauen, 2013). However, some little works are carried out on the utilization of various domestic, municipal, or industrial wastewaters for the large- or pilot-scale exploitations of microalgae cultivations in raceway ponds. Therefore, it is imperative to recapitulate those findings and discuss them in a precise manner in this review article. Park and Craggs, in the year 2010, had cultivated the microalga *Pediastrum* sp. in a 31.8 m^2^, 8,000 L volume pilot-scale raceway pond with domestic wastewater treatment. The raceway pond was paddle-wheel operated with 1 m wide, galvanized steel paddle-wheel circulated inside the raceway pond water to provide the surface velocity of 0.15 m/s. In this study, the areal biomass productivity was recorded 25 g m^−2^ day^−1^ (Table 2). Another study was conducted with a consortium prepared with 15 native microalgal strains, successfully cultivated in a 3,800 L capacity outdoor raceway pond. This was done by utilizing the industrial wastewater containing 85–90% carpet industry effluents with 10–15% municipal sewage wastewater. The culture was supplemented with 6% CO_2_ sparging, and the overall annual biomass productivity was calculated as 9.2–17.8 tons ha^−1^ year^−1^ (Chinnasamy et al., 2010). Continuation to this, the 8,000 L capacity raceway ponds were used for the cultivation of the mixed microalgal consortium as *Scenedesmus*, *Chlorella*, *Pediastrum*, *Nitzschia*, *Cosmarium*, and other filamentous microalgae. The ponds were fabricated in the wastewater treatment plants’ location, and the effluents of the plants as wastewaters were utilized followed; the average areal biomass was found to be 13.5 g m^−2^ day^−1^ (Lee et al., 2014). It is here worth mentioning that apart from the biomass and lipid productivities reported in this study, the other important part can be incorporated to note that the microalgal biomass harvesting process were made much easier and cheaper in those raceways with the use of some mesh like substrates attached with the microalgae, which were simply removed from the microalgal cultures and the treated wastewaters can be discharged more easily. The green microalga *Chlorella vulgaris* was also grown in the huge-sized, actual large-scale raceway ponds with a full capacity of 14.62 billion L with the supply of various wastewaters available from different sources. The culture depth was set as 30 cm for all studies (Table 2). In a contrary to these study reports published, Hena et al. (2015) showed that the annual biomass and lipid productivities could be achieved up to >150 and >25 tons ha^−1^ year^−1^, respectively for the microalgal consortium, isolated from wastewaters and also grown in wastewaters, collected from dairy farms, under the semi-large scale raceway pond cultivation with the working volume of 600 L of one pond (Table 2). The supplementation of 10% CO_2_ was provided for the elevated growth rate of the microalgae. The raceway pond’s dimension was set as 2.5 × 0.7 × 0.7 m, and the mixing speed of the paddle-wheel was fixed at 20 rpm throughout the experimentations. It is indeed highly curious to critically observe this research work’s results as it demonstrated an exceedingly high annual biomass and lipid productivities claimed that are incomparable to many studies reports published till date for microalgal biomass and lipid production under large- or semi-large-scale raceway pond cultivations.

**TABLE 2:** Review on biomass productivity of various microalgal species with the utilization of wastewaters as growth medium under raceway pond cultivation.

Test organism	Cultivation description	Biomass productivity (areal/annual)	References
*Pediastrum* sp.	Raceway pond microalgal cultivation with domestic wastewater treatment, raceway pond dimensions: surface area: 31.8 m^2^, depth: 0.3 m, 8,000 L of raceway pond working volume	Areal productivity: 25 g m^−2^ day^−1^	Park and Craggs (2010)
A consortium of 15 native microalgae	Raceway ponds had of total 3,800 L capacity, wastewater containing 85–90% carpet industry effluents with 10–15% municipal sewage	Annual productivity: 9.2–17.8 tons ha^−1^ year^−1^	Chinnasamy et al. (2010)
Mixed microalgal consortium	Raceways were constructed in the wastewater treatment plant site. 8,000 L; Area- 21 m^2^	Areal productivity: 13.5 g m^−2^ day^−1^	Lee et al. (2014)
*Chlorella vulgaris*	Volume - 14.62 billion L, 30 cm culture depth; alga cultivation utilizing wastewaters	Areal Productivity: 15 g m^−2^ day^−1^	Rogers et al. (2014)
A consortium of microalgal isolates collected from wastewaters	Growth medium: wastewaters from dairy farms; Dimensions: 2.5 × 0.7 × 0.7 m, working volume: 600 L	Annual productivity: 153.54 tons ha^−1^ year^−1^	Hena et al. (2015)
*Chlorella pyrenoidosa*	Growth medium: domestic wastewater; raceway dimension: 1.5 × 0.6 × 0.4 m, raceway working volume: 360 L	Areal productivity: 36 g m^−2^ day^−1^	Dahmani et al. (2016)
Mixed microalgal consortium including *Chlorella* sp., *Scenedesmus* sp., and *Stigeoclonium* sp. (CSS)	Growth medium: municipal wastewater; 0.4 ton working capacity high rated raceway pond, optimized culture depth: 20 cm	Areal productivity: 6.16 g m^−2^ day^−1^	Kim et al. (2018)
*Parachlorella* sp. JD076	Semi-continuous operation in municipal wastewater under small-scale raceway pond cultivations	Areal Productivity: 22 g m^−2^ day^−1^	Yun et al. (2018)
*Desmodesmus subspicatus*	Wastewater mediated algal growth in 2,000 L raceway ponds (2,000 L × 4 raceways)	Areal Productivity: 28 g m^−2^ day^−1^	Schneider et al. (2018)
Mixed microalgal consortium	High rated algal raceway lagoon (length - 30 m and width of the single channel - 2.5 m), community wastewater utilized.	Areal Productivity: 31.7 g m^−2^ day^−1^	Buchanan et al. (2018)
*Chlorella* spp.	Oblong shallow raceway pond having total area was 3.6 m^2^, culture depth was set as 40 cm and total height was 50 cm	Areal Productivity: 2.5 g m^−2^ day^−1^	Romagnoli et al. (2020)

Nonetheless to the previous, there are some other reports which have signified the feasibility of utilizing the wastewaters for microalgal cultivation and simultaneous bioremediation of the wastewaters by the algal bodies. In Algeria, the green microalga *Chlorella pyrenoidosa* was successfully cultivated in the raceway ponds constructed in the desert area at the domestic wastewater treatment plant site. The length, breadth, and depth of the raceway were 1.5, 0.6, and 0.4 m, respectively, with the culture working volume of 360 L. The cultures were circulated inside the raceway pond using the paddle-wheel powered by a 70 W electric gear motor. In this experiment, the maximum areal biomass productivity was recorded as >35 g m^−2^ day^−1^ (Dahmani et al., 2016). The ability of the microalga for bioremediation, various parameters like chemical oxygen demand (COD), NH_4_ –N, and TP were measured in the course of the cultivation periods, and their average removal efficiencies were reported as 78, 95, and 81%, respectively (Dahmani et al., 2016). Ammonium, and total phosphate are the primary source of nitrogen and phosphorous in wastewater, and controlling its toxic effects are the foremost challenge in wastewater treatment (Yu et al., 2019). In this direction to bioremediate ammonium, nitrate, nitrate, and total phosphate in wastewaters, the locally isolated microalga Chlorella sp. was cultivated in a fabricated outdoor wetland under the mixotrophic cultivation techniques using the piggery wastewaters. Various significant parameters for this wetland cultivation were investigated, such as the aeration rate, nutrient removal by the alga from wastewaters, biomass yield, and the fatty acid methyl esters (FAMEs) compositions. The maximum biomass productivity was recorded as 79.2 mg L^−1^ d^−1^ and the total nitrogen (TN), phosphorus (TP) removal efficiencies were found to be 80.9 and 99.2% (Lee and Chen, 2016), which was much higher than the overall chemical oxygen demand (COD) value depicted as 74.5%. The best cultivation temperature was found as 25°C.

Schneider et al. (2018) recorded a maximum biomass production of 1.12 g L^−1^ which is equivalent to 28 g m^−2^ day^−1^ from the microalga *Desmodesmus subspicatus* grown with wastewaters in 8,000 L volume raceway ponds. The interesting fact was that the wastewater sample was collected from one university’s toilets after one-time treatment with the up-flow anaerobic sludge blankets (UASB), which are the reactors. Another study report also claimed a significantly elevated areal biomass productivity of >30.0 g m^−2^ day^−1^ (Buchanan et al., 2018) for the consortium of mixed algal bloom cultivated in the high rated large-scale raceway lagoons of 30.0 m in length. These facultative raceway ponds were fed with general community wastewater and septic tank effluents (Table 2). In 2019, Eladel et al. (2019) recorded a maximum biomass productivity of 0.073 g L^−1^ day^−1^ from *Chlorella sorokiniana* isolated from local municipal wastewater. Moreover, algal growth for 10 days in municipal wastewater depicted a nutrient removal efficiency of 74.20, 83.31, and 78.00% for NO_3_^–^, NH_3_, and total phosphate, respectively. *Scenedesmus obliquus* was proved to be a potent organism in pilot-scale artificial wastewater processing with 96% removal of ammonia content (Liu et al., 2019). Another recent study reported that about 94 and 66% of NH_4_^+^ and PO_4_^3-^ were removed from wastewater medium using microalgae immobilized on agar (Hu et al., 2020). In continuation, the oleagenous microalga *Chlorella* spp. was successfully cultivated using the wastewater obtained from digestate from biogas plants in a raceway pond having length (L):width (W) equal to 2:1, total area was 3.6 m^2^. The culture depth was set as 40 cm and total height was 50 cm. The areal biomass productivity was calculated as 12.5 g m^−2^ day^−1^ with a growth yield of 0.25 g L^−1^ obtained in just 08 days of cultivation period (Romagnoli et al., 2020). From the various earlier studies discussed above in detailed, it is well comprehended that the microalgae are not only capable to thrive under open pond cultivations using wastewaters as their growth medium, but also these tiny microorganisms are the best potential candidates for the harmful nutrient removals from the primary treated wastewaters.

It is highly imperative to investigate the feasibility of culturing some oleaginous as well as halophilic or halotolerant and acidophilic microalgal strains to bioremediate wastewaters generated from heavy industries like mining, iron/steel, coal, tanning. The microalgae may offer a favourable and unconventional alternate to traditional and conventional technologies in the treatment of heavy metals like arsenic, copper, cadmium, chromium, and lead which are generally present in the industrial wastewater samples originated from heavy industries (Uberoi, 2003). These heavy metal ions can also cause diabetes, cancer, anemia, osteomalacia, and many neurotic or nephrotic syndromes (Lefebvre et al., 2006). However, the execution is not as easy as it sounds. Researchers reported that the heavy industrial wastewaters were well characterized by their high alkalinity, resulting in a pH value of ∼8.0 due to these heavy chemicals used in the technological processes. They have also recorded that the total dissolved solids (TDS) concentrations of the industrial wastewaters are up to the elevated level of 37.0 g L^−1^. In contrast, the suspended solid concentrations were measured as 5.3 g L^−1^ (Leta et al., 2004). Hence, pragmatically comprehend and perform these experiments are major critical tasks by the researchers for evaluations and commercialization aspects (Kongjao et al., 2008).

#### Economic Feasibility of Using Wastewater for Microalgal Cultivation

Nowadays, mass- or large-scale cultivations are essentially needed for the algal biofuel industries, and still, several tailbacks are limiting the establishment of commercial level algal bio-oil plants (Grobbelaar, 2012). Microalgal biofuel has gained a tremendous impetus as an alternative to the conventional fossil fuels but the economic feasibility is still a big hindrance for its commercial acceptability throughout the world. One of the several innate challenges is its high cultivation cost. The most acclaimed strategies for the large-scale microalgae productions are the algal growth in raceway ponds that are so termed because of their raceway like shape (Prussi et al., 2014). However, several researchers have addressed that the biggest bottleneck for the microalgal cultivations in the open raceway ponds is the high costs of the chemical-grade growth medium (Kumar et al., 2015; Bhattacharya et al., 2016; Karthikeyan et al., 2016). Nowadays, the microalgal cultivation utilizing the wastewaters in raceway ponds seems to be the preeminent solution in this regard. Wastewater is a cost-effective solution and have already proved as an alternative to the cost incurring algal growth medium (Wu et al., 2014). Since the last decade, microalgae cultivations using various mixotrophic techniques have been practiced and an increased interest in implementing them as part of wastewater treatment coupled with low-cost biofuel generation using the algal slurry. The algae have the potential to utilize the inorganic and organic carbon sources in wastewater bodies vis-à-vis can utilize the inorganic nitrogen and phosphorous particles in wastewaters. From the above discussion, it is clear that various studies have reported that the microalgae can bioremediate more than 90% of the initial nitrogen (ammonia, nitrate, nitrite) and phosphorous (phosphate) from wastewaters. The cost of the produced bio-oil can also reduce substantially using the wastewater where algae can able to thrive even it is highly polluted with nutients and other particles (Mohsenpour et al., 2021).

**FIGURE 1:**
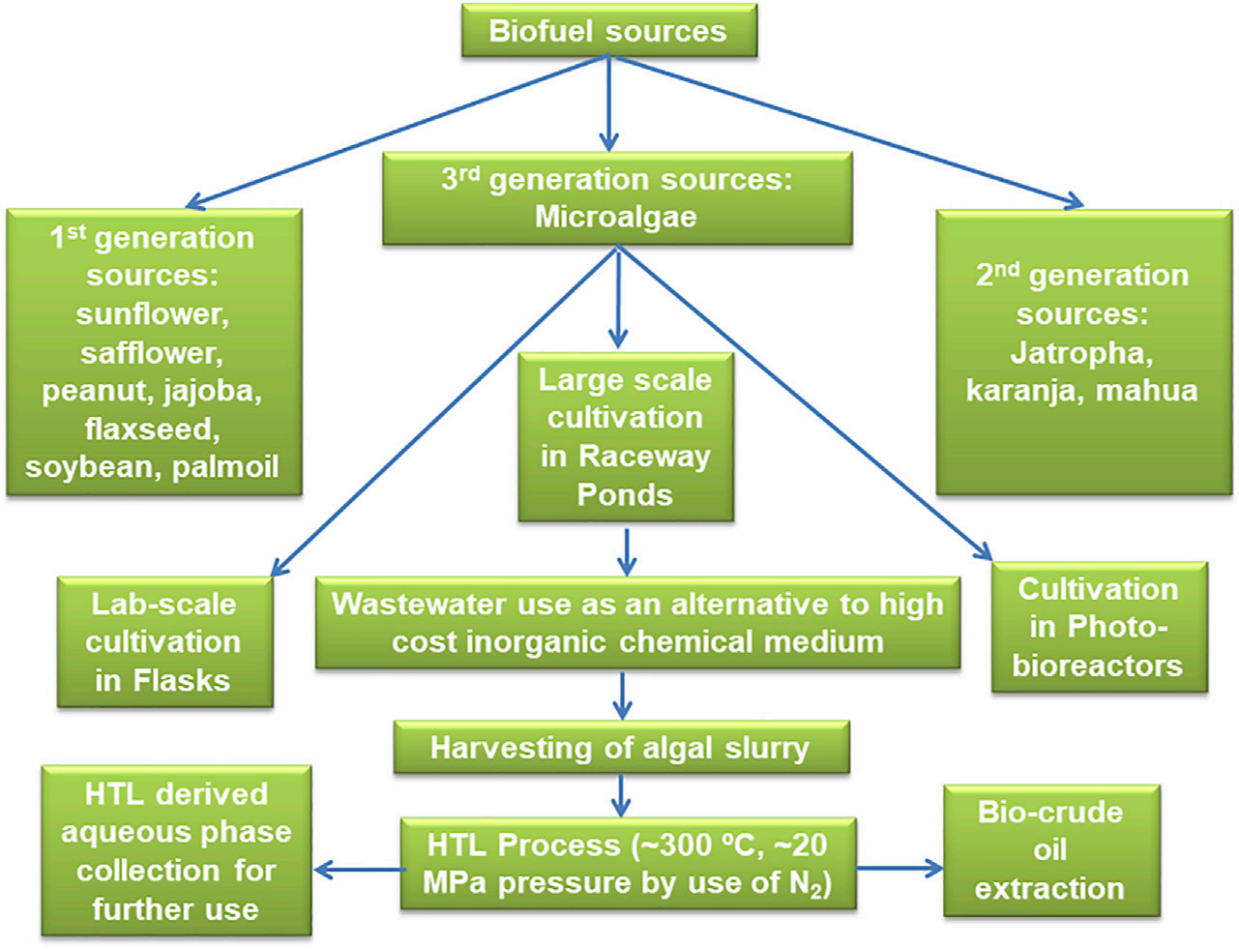
Schematic illustration of microalgal biodiesel production techniques focuses on large-scale cultivation strategies in raceway ponds and direct biomass processing to oil using hydrothermal liquefaction.

Moreover, if we look from the industrial and commercial point of view, nowadays it is undoubtedly the most indispensable to follow some more efficient techniques by which the wastewater grown wet algal biomass can directly be converted into bio-crude oil without adopting or involving the numbers of cost-intensive and time-consuming processes essential for biodiesel production such as dewatering, drying, lipid extraction with solvents, followed by the transesterifications. On a serious note, it can be commented that those lengthy conventional techniques are the real stumbling blocks for biodiesel production in the commercial scales. It is also a clear fact that microalgal biodiesel production is practically an energy and cost-intensive approach due to these lavish and time-consuming harvesting, drying, and solvent-mediated lipid production techniques (Mathimani and Mallick, 2018) (Figure 1). These two steps harvesting and drying incur a substantial economic bottleneck, for higher energy consumption and lengthy time duration. Scientists have strongly suggested to use alternative thermochemical techniques to produce the bio-crude oil directly from the wet microalgal slurries using thermochemical conversion process (Aliyu et al., 2021).

## Approaches Towards the Effective Harnessing of Bio-Crude Oil Using Hydrothermal Liquefaction Technique

### Prominence of Hydrothermal Liquefaction Process

Since the last two decades, various methods were practiced to efficiently cultivate microalgae under large scale biomass generations and numerous implementations and/or modifications of several techniques associated with the different down-streaming processes like harvesting, drying, lipid extractions, transesterifications, and biodiesel production. However, there are minimal new approaches to make the algal biofuels commercially viable and ready to market available with a quick processing and cost-effective manner. Because of these facts, a recent trend has been followed by utilizing various thermochemical conversion techniques that are comparatively economically worthwhile (Chen et al., 2015). Thermochemical conversions of algal biomass to biofuel in the form of liquid or gas is generally useful with the applications of various techniques such as pyrolysis, direct combustion, torrefaction, gasification, and liquefaction by the involvement of different catalysts with the elevated temperatures (Kumar et al., 2017; Kumar et al., 2018).

The most common process for the thermochemical conversion is pyrolysis, which is involved in the thermochemical decompositions of organic matters into energetically useful and condensed liquids, solid residues, and a mixture of gases in the absence of oxygen and the absence or presence of catalysts (Morgan et al., 2017). However, the liquefaction technique is possibly the best suited for the direct conversion of algal biomass to bio-oil. It offers some energetic gains than the other alternative methods, such as pyrolysis, by using wet algal biomass and relatively effectual products’ separations. Remarkably, the bio-crude properties obtained through all of these thermochemical conversion techniques are essentially dependent on the algal biomass feedstock’s quality in carbon and hydrogen and must be low in nitrogen, sulfur, oxygen, and ash contents (Cole et al., 2016).

HTL is the technique to convert wet algal feedstocks (∼90% moisture content wet basis) directly into bio-crude oil at the elevated temperature and pressure of ∼200–600°C and 10–25 MPa, respectively in the presence/absence of some catalyst with a typical processing time of 10–100 min, depending on the technological efficiencies with their practical implementations (Biller and Ross, 2011; Zhou et al., 2013; Couto et al., 2018). It is indeed an interesting fact that the bio-crude oil extracted with the HTL applications is generally higher than the overall lipid content of the microalgae because the proteins and carbohydrates of the algae may also be converted into oil under the elevated pressure and extreme higher temperature required for HTL technique (Cheng et al., 2018). Therefore, a wide range of algae biomass can be converted into crude oils, and it may be commented that the HTL technology is best suited for the outdoor, raceway ponds’ cultivated microalgal biomass processing just after the harvesting. Furthermore, HTL also resolves the issues of energy balance for biofuel production process as water along with the catalysts serves as the reaction medium for this technology (Zhou et al., 2013) evades the requirements of the drying processes which alone generally needs 30% of the total production costs of biomass to biodiesel (Becker, 1994). The most advantageous part of the HTL process is that the aqueous wastewater which is self-seperated and is generated after obtaining the bio-crude oil, can be collected to reuse as the growth medium for the microalgal cultivations (Peterson et al., 2008; Cheng et al., 2017). Nonetheless, it was quite frequently observed that the microalgal lipids might be hydrolyzed and converted into the free fatty acids at a temperature below 250°C, required for the proper operation of HTL process. Further increase in the reaction times and temperatures, the algal cell walls generally break, and the carbohydrates and proteins may undergo deamination, decarboxylation, or re-polymerization (Neveux et al., 2014).

The foremost requirement in the HTL technique is that the microalgal feedstock biomass should not contain a high level of nitrogen, which is not recommended to produce the qualitative bio-crude oil. The elevated nitrogen content in the biomass is not technically feasible for purifying the bio-crude oil using catalysts (Mehrabadi et al., 2015). The bio-crude oil, rich in nitrogen, oxygen, or sulfur, may necessitate ample up-grading with hydrogen before starting a normal purifying process; thereby, the cost-effectiveness and energy inputs for the bio-oil production process will be much higher. However, it is a fact that the microalgal biomass usually contains 3–6% of internal nitrogen, which is stored for use in future times of cellular proliferation. Therefore, it is essential to modulate and lowering the internal nitrogen content of algal cells at the ending of the cultivation periods by the proper management and manipulations of the culture conditions in open raceway ponds before harvesting the wet biomass directly for the bio-crude oil production using HTL process (Duan and Savage, 2011). This major problem was successfully resolved by a recent technique demonstrated by the research work of Cole et al. (2016) in which some suitable organic non-polar solvents were mixed with the algal biomass slurry just before the operational separation of HTL process.

In hydrothermal conversion technique, the biomass of the algae is changed by the extreme hot and compressed water into comparatively shorter carbon chains that have a higher saturation, and thereby the energy values are also relatively higher (Brennan and Owende, 2010). The process’s main product contains the heavy bio-crude oil comprised of C16–C18 hydrocarbons, and the crude oil yield from this process is approximately 30–50% of the dry weight (dw) with a heating value in the range of 30–40 kJ/g. The major by-products from this HTL process are the gaseous mixtures, which contain carbon di-oxide, hydrogen, methane, nitrogen, ethane, and acetylene with some residual solids less than 10%wt, and an aqueous phase with 20–30 (wt%) yield (Mehrabadi et al., 2015). The main advantage of considering the HTL technique for large-scale cost-effective biofuel production from algae is that the aqueous phase obtained in the HTL process generally contains a higher amount of essential and major nutrients. This could be recycled again for the microalgal cultivations by taking the aqueous phase mixtures as wastewater sample.

### Analysis of Various Studies of Catalytic and Non-Catalytic Hydrothermal Liquefaction Reactions

**FIGURE 2:**
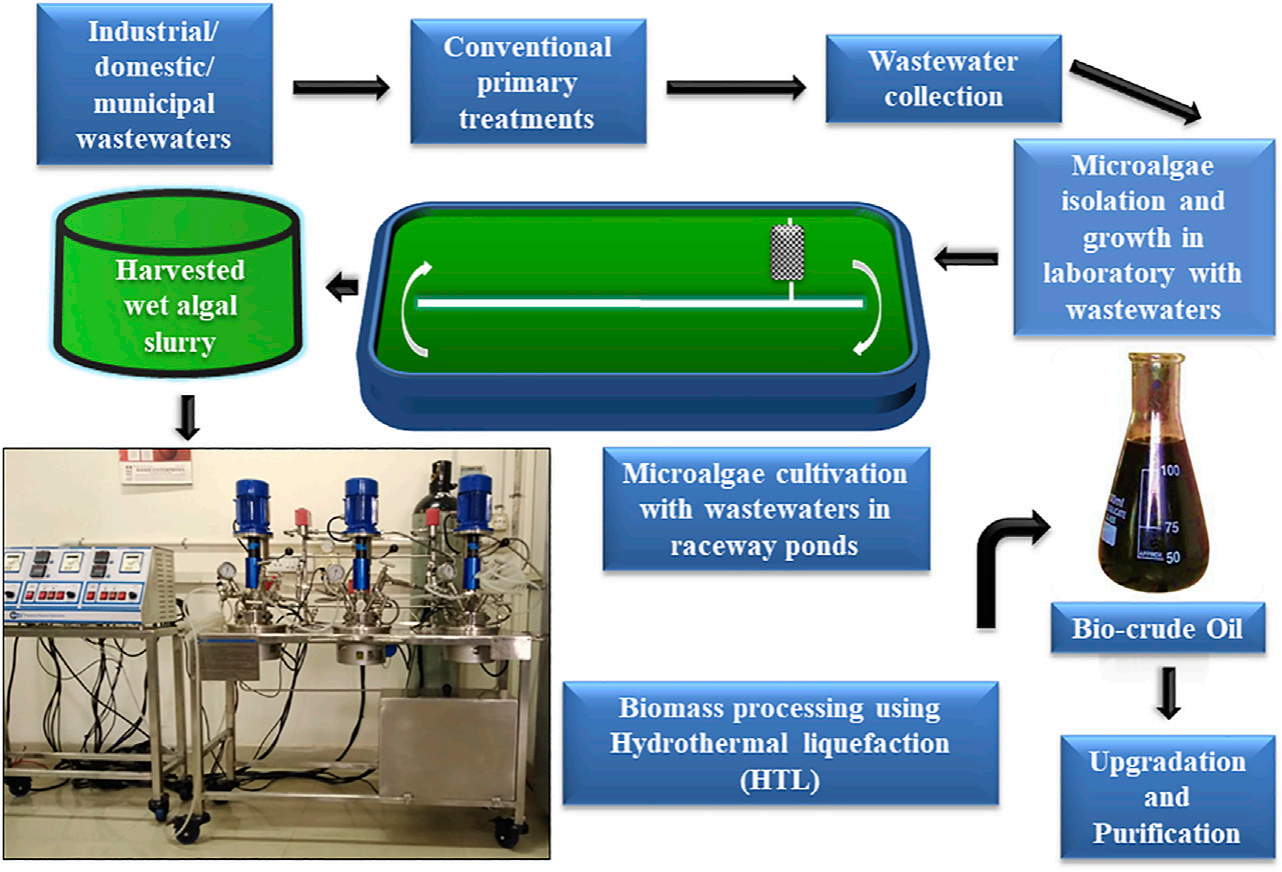
Diagrammatic representation of microalgal cultivation in large-scale raceway ponds with wastewaters coupled with bio-crude oil production using wet algal biomass hydrothermal processing.

**FIGURE 3:**
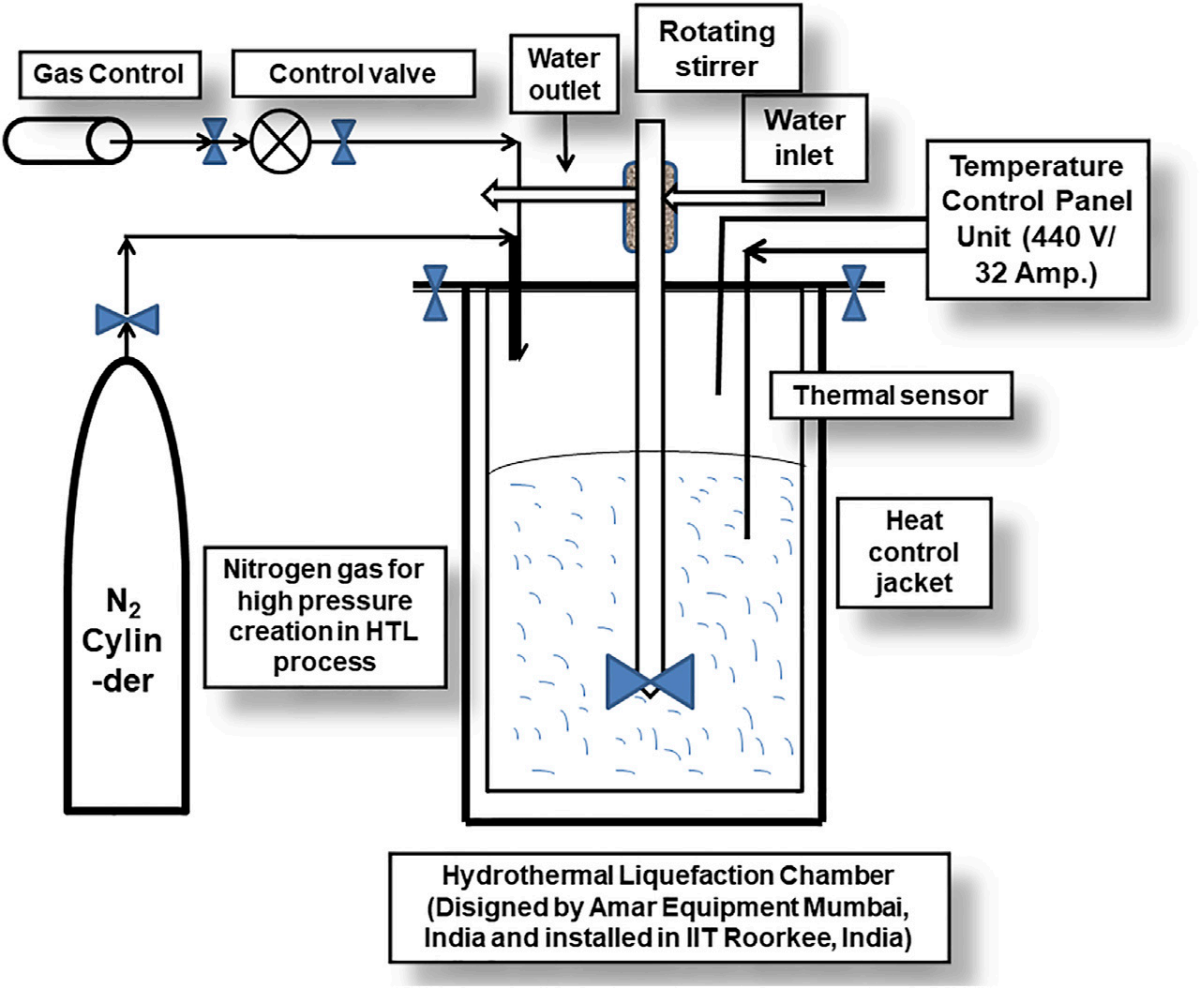
Schematic diagram of a hydrothermal equipment (batch reactor designed by Amar Equipments Mumbai and installed in IIT Roorkee) highlighting its different parts/componants.

HTL of the wet algal biomass, generated from the large-scale raceway pond cultivations of microalgae by utilizing the industrial or domestic wastewaters are seemed to be the best promising approach towards the cost-effective production of the renewable and sustainable biofuel, replacements to the conventional fossil fuels. In concern of the large-scale exploration of the wet microalgal biomass processing, HTL is one of the most superior technologies for converting biomass to bio-oil, bypassing the energy and cost-intensive processes like dewatering, drying, and solvent-mediated lipid extractions (Figure 2). Several recent works are done for crude bio-oil production from microalgae using different catalysts or without any catalysts under hydrothermal explorations. In 2011, Biller and Ross utilized the HTL technique for the two chlorophycean microalga *Chlorella vulgaris* and *Nannochloropsis occulata* at a temperature and holding time of 350°C and 60 min, respectively. In another study, the bio-crude oil was produced from the alga *Nannochloropsis* sp., with the reactions in water at a temperature of 350°C and holding time of 1 h and in the addition of various heterogeneous catalysts such as Pd/C, Pt/C, Ru/C, Ni/SiO2-Al2O3, CoMo/*γ*−Al2O3, and Zeolite. The maximum bio-oil production and the heating value were depicted as 57.0 (wt%) and 38.0 MJ kg^−1^, respectively (Duan and Savage, 2011). The bio-crude oil yield reported in this research process is incredibly higher than many other research works on bio-oil yield by using the HTL technique, published to date. Contrary to using the high-cost catalysts for reactions in HTL technique, researchers proved that the overall oil yield was achieved up to ∼45 (wt%) without any catalysts that can act as stimuli. The mixed microalgal consortium was heated at 350°C for 60 min under HTL reactions without any catalyst addition, and the maximum bio-crude oil yield and heating value (% of energy recovery) were shown as 44.5 (wt%) and 39.0 MJ kg^−1^ (Roberts et al., 2013). The bio-oil yield primarily depends on the HTL process temperature, catalyst used, the solvent used, reaction time. Researchers have found exciting findings that the carbohydrates in the algal biomass can rearrange to aromatic compounds, and polymers are converted to monomer units. In contrast, the proteins components are restored to pyrrole, and some other amide compounds during the time course of HTL reaction (Raheem et al., 2018). Figure 3 illustrates a schematic representation of typical HTL reactor installed in IIT Roorkee.

Some synergistic approaches of the large-scale raceway pond cultivation coupled with direct bio-oil production from the wet algal biomass was successfully performed for the unbranched and filamentous, chlorophycean microalga *Oedogonium* sp. under the large-scale cultivation in a recirculating aquaculture system consisting of six 10,000 L parabolic raceway ponds with the dimensions of 7.1 m length, 2.2 m width, and 71.0 cm in depth. The wet algal biomass was taken for direct HTL technique at the temperature and pressure of 350°C and 180 bar, respectively. The catalyst was chosen as Ni2P/SiO2, and the maximum crude oil yield was found to be 22–23 (wt%), whereas the heating value (% energy recovery) was 22.0 MJ kg^−1^ (Cole et al., 2016). Though it seems that the bio-crude oil yield was relatively low in this process, the most significant part of the research work was the overall time requirement, i.e., only 3 min, which is relevant in cost-effective bio-oil production (Table 3). In accordance, one recent work has demonstrated that the maximum bio-oil yield was 44.4 (wt%) and the heating value (% of energy recovery) was 38.1 MJ kg^−1^, respectively, at a reaction temperature of 300°C without the addition of any catalysts in the conversion for the hydrothermal process. This study report has also depicted that the overall conversion time for the HTL process was found as 15 min only (Couto et al., 2018). The research work performed by Koley et al. (2018) have shown that the biomass of the green microalga *Scenedesmus obliquus* was hydrothermally processed to obtain the bio-oil varying the temperature and pressure ranges. However, the best suitable temperature was found to be 300°C and pressure of 200 bars with a conversion time of 1 h. The study revealed that the harvested microalgal biomass contained high oxygen and carbon presence of 36.1 and 48.1%, respectively. The crude oil content was enhanced as 45.1 (wt%) with the addition of catalyst as CH_3_COOH compared to bio-oil content of only 35.7 (wt%) for no addition of any catalysts. The bio-crude oil content was gained up to the maximum value of 65.7 (wt%), which is significantly higher under the HTL process application for microalgal bio-oil production, reported in other studies to date. In that conversion technique, the most suitable reaction temperature and processing time were recorded as 350°C and 1 h, respectively, with the catalyst as CuO/Al-SBS-15, utilized to fasten the conversion process the HTL chamber (Jing et al., 2018) (Table 3). In continuation, Xu et al. (2018) has also experimented with the HTL technique for the wet algal slurry using various catalysts at a temperature of 350°C for 20 min. In that course of studies, various combinations of catalysts were tested for the efficient bio-crude oil yield viz., no catalysts (none), 10% Ni 0.1Ru/CeO2, and 10% Ni/CeO2. The HTL process was further upgraded to a temperature of 450°C for 1 h with a maximum HTL pressure of 225 bars, and the bio-crude oil yield was found to be 57.14 (wt%) with the addition of Ni-Ru/CeO2 + H2 catalysts. The maximum heating value/% energy recovery has also recorded a value of about 40 MJ kg^−1^. However, there are also reports available for the lower bio-crude oil production using this HTL process. It was optimized that the maximum crude oil production was only 18.0 (wt%) under the reaction temperature of ∼300°C operated at a very low pressure of 60 bars (6 MPa) with no use of catalysts. Interestingly, the microalga *S. quadricauda* was cultivated in the outdoor large-scale open raceway ponds with some other species of *B. braunii* and *C. vulgaris*. The GC-MS analysis of the produced bio-oil confirmed the presence of various organic and fatty acid esters, some nitrogenous and oxygenous compounds, alkanes, and hydrocarbons (Kiran Kumar et al., 2018) (Table 3). In a very recent study, it was found that the bio-oil yield was 26.0 (wt%) utilizing HTL technique for demineralized wastewater algal biomass. The wastewater was collected from an wastewater treatment plant in a swine farm at the University of Illinois Urbana-Champaign. The optimized reaction temperature was recorded as 280°C for 1 h reaction time. Moreover, the GC-MS study revealed that the bio-oil was rich in hydrocarbons and found comparable with the fuel properties of various international levels (Carpio et al., 2021).

**TABLE 3:** Reviews of various recent experimental reports on catalytic/non-catalytic hydrothermal liquefaction process for bio-crude oil production.

Microalgal species	The temperature of HTL process (°C)	Holding time (min)	Catalyst used	Bio-crude yield (wt%)	Maximum heating value (MJ kg^−1^)	References
*Chlorella vulgaris*	350	60	No catalyst	38.0	54.2	Biller and Ross (2011)
			1 M Na_2_CO_3_	28.0	44.2	
			1 M HCOOH	28.0	31.7	
*Nannochloropsis occulata*			No catalyst	36.0	66.1	
			1 M Na_2_CO_3_	26.0	50.0	
			1 M HCOOH	28.0	41.1	
*Nannochloropsis* sp.	350	60	Pd/C; Pt/C; Ru/C; Ni/SiO_2_-Al_2_O_3_; CoMo/γ-Al_2_O_3_/Zeolite	57.0	38.0	Duan and Savage (2011)
Mixed microalgal consortium	350	60	No catalyst	44.5	39.0	Roberts et al. (2013)
*Nannochloropsis oceanica*	300	30	No catalyst	40.1	36.3	Cheng et al. (2014)
*Chlorella pyrenoidosa*	300	60 13 MPa pressure	NaY, USY, HY	64–68	Not reported	Yang et al. (2016)
*Oedogonium* sp.	350	30	Ni_2_P/SiO_2_	22–23	22.0	Cole et al. (2016)
*Chlorella* sp.	300	30	9 MPa, 10% biomass loading, no catalyst	32.5	∼34	Reddy et al. (2016)
*Galdieria sulphuraria* CCMEE 5587.1	350	60	Not specified	30.8	Not reported	Cheng et al. (2017)
*Nannochloropsis salina* CCMP 1776	310			59.1		
*Aurantiochytrium* sp. KRS101	400	10	Not specified	51.2	∼33.0	Vo et al. (2016)
*Cyanidioschyzon merolae*	300	30	12 MPa, 10% biomass loading, Catalyst 0.5 M KOH/NaOH	22.7	33.7	Muppaneni et al. (2017)
Microalgal consortium	300	15	No catalyst	44.4	38.1	Couto et al. (2018)
*Scenedesmus obliquus*	300	60	No catalyst	35.7	35–40	Koley et al. (2018)
			CH_3_COOH	45.1		
*Chlorella* sp.	∼350	60	CuO/Al-SBA-15	65.7	Not reported	Jing et al. (2018)
*Nannochloropsis* sp.	450	60	Ni-Ru/CeO_2_ + H_2_	57.1	∼40	Xu et al. (2018)
*Scenedesmus quadricauda*	300	30	No catalyst	18.0	Not reported	Kiran Kumar et al. (2018)
*Chlorella pyrenoidosa*	150–300	Each temp. resting at 10 min	2.73 g of Deionized water	33.3	34.5	Obeid et al. (2019)
Mixed algal culture	280	60	Not specified	26	∼35	Carpio et al. (2021)

## Major Advantages and Drawbacks of Hydrothermal Liquefaction Process

HTL process has certain disadvantages despite being the most effective, suitable, and least time-consuming thermochemical conversion process for the large-scale, raceway pond grown with the wastewater mediated cultivation of microalgal slurries be converted directly into bio-oil for ready to market purpose servings. Compared with the conventional biodiesel manufacturing technologies available, the major advantages of the HTL process are found to be a lot. This HTL technique has eliminated the essentiality of considering only elevated lipid yielding microalgae followed by cell disruption, dewatering, drying, and solvent recovery for lipid extraction process (Cheng et al., 2017). The most exciting part of the use of HTL technique is that the large-scale biomass can be processed and converted into the bio-crude oil in a concise time period of only 30–60 min only. This is very beneficial from the industrial point of view to make the algal oil commercially viable. But the major cons of HTL are the necessity of high energy inputs (∼300–500°C) with the elevated input pressure (∼15–20 MPa). As the products after the HTL process have constituted a very high content of nitrogen and oxygen, the product seems to be quite unstable. There are some further upgrading of the HTL products therefore decidedly essential. The upgradation involves converting oxygen to CO_2_ and nitrogen to ammonia (Faeth et al., 2013; Mehrabadi et al., 2015).

Since its inception, algal biomass is utilized for biodiesel production from lipids using transesterification. However, researchers also tried to harness the biodiesel from lipids during its first phase. It simultaneously generated the bio-crude oil from the residual biomass using HTL technique during its latter stage utilizing the carbohydrates, remaining lipids, and proteins from the same microalgal biomass. The competency of using the defatted biomass after lipid harnessing has a significant impact on the total energy equilibrium for algal biomass to the biofuel production process (Xu et al., 2011). In order to exploit the biomass residues remaining after the lipid harnessing, bio-crude oil can efficiently be generated from the defatted algal biomass residues including carbohydrates and proteins. However, minimal approaches are made in this direction (Cheng et al., 2014), which are actively produced biodiesel in its former stage and co-generated the bio-crude oil in its later stage using HTL technique. Moreover, it is also interesting that a part of the energy recovery of HTL process may also be possible by using the outflow of the HTL chamber to heat the inflow with a convincing positive net energy balance. The aqueous phase that contains the ammonia and specific nutrients can be recycled and may be used as an agricultural fertilizer like the biochar.

The biggest stumbling block for the HTL technology is that it still not proceeding on a commercial scale for cost-effective production of bio-crude oil, so the advantages somewhat seem to be more theoretical (Yang et al., 2014; Smith and Ross, 2016). It is also noticed that the bio-crude oil is composed of various fatty acids, amides, and aliphatic molecules, whereas; a part of the bio-oil contains more nitrogen and oxygen heteroatom aromatic components. For large-scale biomass processing in HTL, these heteroatom nitrogen and high molecular weight containing large molecules in the bio-crude oil are the major concerns for upgrading bio-crude oil (Elliott et al., 2015). These high N-containing and high molecular weight components might be generated from the elevated protein and carbohydrate comprising microalgal biomass to bio-oil conversion process. Nevertheless, researchers have recommended that the pre-treatment processes must remove the carbohydrates for HTL technique rather than the removing of protein components. This is essential because the carbohydrate components can produce some highly aromatic heterocyclic compounds that are very difficult to upgrade in the whole HTL process (Cheng et al., 2017). The above discussions regarding the pros and cons of HTL process evoks that there is a need of design commercial scale hydrothermal equipment which can process a large-scale microalgal biomass in a very short period to bio-oil. Recently, Johannsen et al. (2021) have nicely designed a large-scale HTL batch reactor for processing of biomass. The core part of the HTL plant is equipped with 58 type-K thermocouples. 32 of these are located in the trim heater measuring the temperature of the individual heat clamps. The rest are located along the 147 m pipe system, approximately 6 m apart, ensuring a detailed overview of the temperature profile. The main unit is also outfitted with a trim heater, reactor, cooler, thermocouples, and heat exchangers. This large-scale HTL plant made up with the polycarbonate coffer and inner protective steel and it has the active suction from all areas (Johannsen et al., 2021). This kind of experimentations are highly needed for pilot plant set up with HTL technique with which the large-scale algal slurry can be processed to bio-oil in a timely manner.

## Utilization of Hydrothermal Liquefaction Aqueous Phase and Use of Biochar

Hydrothermal processing is gaining immense importance for biomass processing, starting from lignocellulose feedstocks to the tiny algae for crude oil production (Gai et al., 2014; Guo et al., 2015; Zheng et al., 2017). However, the HTL process applications for the wet algal biomass grown under the wastewater mediated large-scale microalgae cultivation have not yet been fully explored to date. It is also interesting that the hydrothermal aqueous phase can be reutilized as a wastewater source for the outdoor cultivation of microalgae. This aqueous phase which is derived as a huge quantity at the end of the HTL process, is generated due to the elevated moisture content (∼90–95% wet basis) of the wet algal biomass (Lee and Chen, 2016; Leng et al., 2018). The aqueous phase is generally comprised of high levels of organic carbon and nitrogen compounds as well as various toxic components viz. some heavy metals and oxygen or nitrogen heterocyclic counterparts (ring structure cyclic compound) like pyrrole and Pyrrolidine. However, a rare study report is for the disposal of this huge quantity of aqueous phase for the re-utilization of it coupling with various wastewaters as the additional growth-promoting nutrient-rich medium for microalgae (Jena et al., 2011; Hognon et al., 2015).

On the other hand, there is an extreme prerequisite of huge quantities of the wastewaters rich in nitrogen and phosphorous for the large-scale commercial level cultivation of microalgae. Nevertheless, only utilizing the aqueous phase derived through the HTL process for the mass cultivation of microalgae may not be able to meet the complete requirement in this case. But the synergistic approach to utilize the aqueous phase of HTL conversion process with the industrial and/or domestic wastewaters as the growth medium for the mass cultivation of microalgae could be the best possible approach towards the cost-effective renewable biofuel production in a commercial scale. There are many reports are available for the reutilization of this aqueous phase for the evaluation of microalgal cultivations (Pham et al., 2013). One study report has already demonstrated the successful outdoor cultivation of microalga *Chlamydomonas reinhardtii* with the hydrothermal mediated aqueous phase wastewater (Becker et al., 2014). However, several researchers performing different studies have confirmed that the aqueous phase generated after the HTL reactions generally was contained various nitrogen, high phosphate ions that are quite beneficial for the algal growth but the existence of the heavy metal ions, phenolic and some furans compounds such as toluene, 2-Methylbenzofuran, and various toxic nitrogenous compounds such as amino-phenol, pyridine, piperidinone were also noticed (Huang and Yuan, 2016; Toufiq Reza et al., 2016). These certain chemical compounds are also inhibitory for the microalgal growth; hence it has a limitation and the proper research should be carried out in this area in the near future (Gollakota et al., 2018). While discussing the recent study reports on HTL process used for algal bio-crude production, it can also be suggested that the processed bio-crude may be used like petro-crude in the petroleum refineries. However, this could be done only after its proper denitrogenation and deoxygenation. It may also be commented that the HTL derived aqueous phase re-utilization with cost-effective hydrothermal processing of algal biomass to biofuel is a very recent and limited approach and thereby, challenges still exist in this field. This can be overcome with future research studies by the technocrats and scientists’ efficient combined and mutual works in this direction. A recent study have focused to use the pulse electric field as the pretreatment of microalgae for the HTL process. This pretreatment method can reduce the final nitrogen content in the biocrude. In another way the steam catalytic cracking technique may reduce the oxygenate level in the bio-crude oil and can enhance the hydrocarbon content (Aliyu et al., 2021).

Biochar, one of the most important co-products of algal biorefinery approach are obtained from the hydrothermally processed algal slurry. One recently published article has focused on the co-carbonization of algae with some different feedstocks to generate nitrogen-doped highly microporous biochar specifically named as hydrochar (Aliyu et al., 2021). With these techniques the last product of HTL after the aqueous phase extraction, i.e. biochar will be safe for agricultural fields with a proper balance of nitrogen, carbon and oxygen content.

## Conclusions and Future Perspectives

Biomass-based energy is the main form of renewable energy today. As per the International Energy Agency and many other national and international organizations, if commitments for global climate-change are to be met with, then, bioenergy has tremendous potential to effectively provide a solution for a low carbon global energy system in the future, specially through decarbonisation of aviation, shipping and road transport sectors. However, currently exploration and exploitation of biomass-based resources for bioenergy production is tragically much below the required quantity needed to be deployed. Under such circumstances, ramping up of sustainable biofuel generation through accelerated usage of renewable resources is pertinent, particularly in the transportation sector where fuel consumption is estimated to triple by 2030. But biofuel is a difficult and slightly complicated topic, specially when it comes to addressing the sustainability index in the low-carbon global society in the future. This review article therefore re-examines the current state of biofuel research worldwide and suggests solutions to overcome the challenges related to lower biomass and oil yield, longer biomass treatment procedures and multi-step oil extraction methods during algal biofuel production. It also provides a workable roadmap through utilisation of biomass-based renewable resources, more specifically the green chlorophycean microalgae grown in industrial untreated wastewater for a one-step bio-oil production using HTL technique. Additionally, it also addresses the problem of water scarcity through re-usage of the processed water after cultivation and/or HTL. Furthermore, such a strategy suggesting the combination of three factors, namely, microalgal cultivation, wastewater bioremediation and HTL technique is not available/very scantily available to the extent of our knowledge. This review article is therefore expected to be extremely beneficial to the readers for cost-effective, environmental friendly algal fuel production on a larger scale for commercial application.

## Declaration of Author Agreement

All the authors listed have approved the manuscript and agreed to authorship and submission of the manuscript for peer review.
